# Challenges in maintaining treatment services for people who use drugs during the COVID-19 pandemic

**DOI:** 10.1186/s12954-020-00370-7

**Published:** 2020-05-06

**Authors:** Adrian Dunlop, Buddhima Lokuge, Debbie Masters, Marcia Sequeira, Peter Saul, Grace Dunlop, John Ryan, Michelle Hall, Nadine Ezard, Paul Haber, Nicholas Lintzeris, Lisa Maher

**Affiliations:** 1 Drug & Alcohol Clinical Services, Newcastle, NSW Australia; 2 Faculty of Health, University of Newcastle, Callaghan, NSW Australia; 3 Drug & Alcohol Clinical Research & Improvement Network, Surry Hills, NSW Australia; 4 John Hunter Hospital, Hunter New England Local Health District, New Lambton, NSW Australia; 5 Penington Institute, Carlton, Victoria Australia; 6 St Vincent’s Hospital, Darlinghurst, NSW Australia; 7 Drug Health Services, Camperdown, NSW Australia; 8 Discipline of Addiction Medicine, University of Sydney, Camperdown, NSW Australia; 9 Drug & Alcohol Services, Sydney South East Local Health District, Surry Hills, NSW Australia; 10 The Kirby Institute, UNSW, Kensington, NSW Australia

## Abstract

The impact of COVID-19 across health services, including treatment services for people who use drugs, is emerging but likely to have a high impact. Treatment services for people who use drugs provide essential treatment services including opiate agonist treatment and needle syringe programmes alongside other important treatment programmes across all substance types including withdrawal and counselling services. Drug and alcohol hospital consultation-liaison clinicians support emergency departments and other services provided in hospital settings in efficiently managing patients who use drugs and present with other health problems.

COVID-19 will impact on staff availability for work due to illness. Patients may require home isolation and quarantine periods. Ensuring ongoing supply of opiate treatment during these periods will require significant changes to how treatment is provided. The use of monthly depot buprenorphine as well as moving from a framework of supervised dosing will be required for patients on sublingual buprenorphine and methadone. Ensuring ready access to take-home naloxone for patients is crucial to reduce overdose risks. Delivery of methadone and buprenorphine to the homes of people with confirmed COVID-19 infections is likely to need to occur to support home isolation.

People who use drugs are likely to be more vulnerable during the COVID-19 epidemic, due to poorer health literacy and stigma and discrimination towards this group. People who use drugs may prioritise drug use above other health concerns. Adequate supply of clean injecting equipment is important to prevent outbreaks of blood-borne viruses. Opiate users may misinterpret SARS-CoV2 symptoms as opiate withdrawal and manage this by using opioids. Ensuring people who use drugs have access to drug treatment as well as access to screening and testing for SARS-CoV2 where this is indicated is important.

## Introduction

COVID-19 is a global pandemic. SARS-Cov2, the virus that causes COVID-19, attacks the respiratory tract [1]. Data on the case fatality rate continue to emerge, currently, it is 3.61% with wide variation between countries [2], and is higher than seasonal influenza [3]. Older people, men and those with medical comorbidities including chronic pulmonary disease, cardiovascular disease, cerebrovascular disease, diabetes and a compromised immune system have a much higher likelihood of complications including ARDS, renal failure and death. People who smoke or vape tobacco or cannabis products and people who are dependent on opioids and methamphetamine use may also be at increased risk of complications due to the respiratory and pulmonary effects of SARS-Cov2 infection. People who are immune-suppressed, for example, due to HIV infection or other chronic medication conditions, are also at increased risk for SARS-CoV2 infection.

Specific populations both face and present unique challenges. Globally, tobacco use, alcohol and other drugs case an estimated 11.8 million people each year in 2016 [4]. Globally, substance use disorders are estimated to have a prevalence of 100.4 million persons for alcohol, 22.1 million for cannabis and 26.8 million for opioids. 4.2% of all disability-adjusted life years (DALYs) were attributable to alcohol use whilst 1.3% of all DALYS were attributed to other drugs [5]. There are an estimated 15.6 million people who inject drugs internationally [6]. Globally, 17.8% of people who inject drugs live with HIV infection, whilst 52.3% are HCV antibody positive, of whom many will be HCV RNA positive [7]. Compared to the general population, people with substance use disorders (SUD) are more likely to have a higher burden of comorbid medical conditions [8, 9]. They are also more likely to experience social and economic disadvantage, homelessness and residential instability [10, 11], imprisonment [12] and difficulties with transportation and often face considerable barriers to accessing health services, including stigma and discrimination from healthcare professionals [13]. People with severe disease may rotate between emergency departments, withdrawal and other drug treatment facilities, homeless shelters and correctional facilities, raising challenges for prevention, screening, isolation and treatment of COVID-19.

COVID-19 presents significant challenges for this population and for treatment providers for people who use drugs. The anticipated wave of clinical SARS-Cov2 infections will have a high impact on emergency department and acute inpatient services as well as primary care services. For treatment providers, deciding on which components of their services are essential and must continue as a priority during this pandemic and which services are de-prioritised to optimise use of health staff and resources are important and pressing decisions.

Internationally, health services across Australia are working to implement strategies to ensure health service continuity, to be able to manage surges in demand for care, proactively reduce COVID-19 transmission and manage patients with confirmed or potential infection. Ensuring primary care and acute care services are best placed to screen, diagnose and provide treatment for those with COVID-19 infections could require re-orienting of the tasks of alcohol and drug staff. Hence, ensuring emergency departments can assess and refer patients with acute substance use-related problems as part of their clinical presentation is important so they can respond to managing people presenting with respiratory infections.

Treatment services for people who use drugs, and in particular and opiate agonist treatment (methadone and buprenorphine), deserve special attention in the response to the pandemic.

1. Opioid agonist treatment (OAT) should be considered an essential treatment during the COVID-19 pandemic, as significant risks to the community exist with an interruption of the stable provision of opioid treatment. Methadone and buprenorphine are on the WHO list of essential medications [14].
2. Patients on OAT are particularly vulnerable to disruptions caused by a pandemic. Co-occurring health conditions and daily dosing in large clinics may crowd many patients in close proximity on a daily or near-daily basis increasing their susceptibility to COVID-19 infections. Opiate agonist treatment dosing and community pharmacy staff have increased infection risks providing these services.
3. Given the above vulnerabilities, without proactive measures, patients attending treatment services may be more susceptible to develop COVID-19 infections, may be less likely to be tested for SARS-CoV2, have increased difficulty complying with home isolation and may be in living situations where the infection may spread rapidly [15].
4. Surging demands elsewhere in the health system resulting in resource and staff redeployment to support other high priority health services risks disrupting critical harm reduction services that could reverse gains made in the treatment and prevention of HIV and hepatitis B and C. As such, planning to ensure these services remain open and accessible is critical.
5. Substance use counselling and withdrawal services may experience changing demand depending on other factors during COVID-19. Distress may result in some people increasing their substance use and subsequently require treatment (for example, alcohol use may increase). Changes in illicit drug supply may occur due to a range of complex interacting factors, with an increased demand for services. Alternately, some people who use drugs may be less likely to request services during the pandemic, with an escalation of substance use during a time of distress.
6. There may be increased risk of opioid overdose arising from (a) erratic access to methadone or buprenorphine dosing, (b) erratic access to illicit heroin supplies and (c) increased access to take-away doses of methadone/buprenorphine necessitating expansion of take-home naloxone supplies, particularly where treatment services are providing increased take-away doses of OAT.
7. COVID-19 itself has potential to increase overdose risk among people with SUD with chronic lung disease, previously identified as a risk factor for overdose mortality [16], and methamphetamine use may place people at increased risk of pulmonary hypertension [17], a risk factor for COVID-19 complications.

This paper discusses possible changes in demand and capacity to deliver treatment services for people who use drugs during the COVID-19 pandemic and identifies mitigation strategies for ongoing management of these changes and mitigation of related risks.

## Service changes during the pandemic COVID-19

Treatment for people who use drugs is provided in a wide range of settings worldwide including community-based settings (clinics, health care centres, primary care, community pharmacies, ambulatory withdrawal), inpatient settings (hospital consultation-liaison services and inpatient withdrawal) and residential settings (withdrawal and rehabilitation). Internationally, services may be operated by state or national health services, non-to-profit organisations or private providers. In some jurisdictions, travel over long distances may be involved for people accessing care.

### Opiate agonist (methadone and buprenorphine) treatment

Opiate agonist treatment requires significant resources due to the known risks of non-medical use of methadone including injection and overdose. Apart from buprenorphine treatment in the USA and France, many other countries’ opiate treatment system is based on supervised daily dosing as default, both for methadone and buprenorphine, with non-supervised or take-away doses being provided to stable patients assessed as being at reduced risk of non-medical use of these medications.

Given the need to provide treatment in many countries where home isolation is now very critical, planning alternatives to daily supervised dosing is important and imposes a major challenge. This is the case since daily supervised opiate treatment may involve significant waiting periods for patients, including people having to wait in queues for extended periods of time; and social distancing may not be practical due to the size of waiting areas and the number of patients.

Considering these issues and the availability of new long depot formulations, key strategies to best utilise resources during the COVID-19 pandemic is impacting on health services are the following:

- Providing depot buprenorphine (e.g. Buvidal weekly and monthly and Sublocade) rather than daily supervised sublingual buprenorphine dosing or methadone dosing;
- Providing additional take away (non-supervised) doses of both buprenorphine and methadone.

There are important safety advantages of buprenorphine over methadone with regards take away or non-supervised doses, as has been seen in France compared to other countries in Europe [18]. Ideally, buprenorphine may be provided as buprenorphine-naloxone to minimise use by injection. While providing additional methadone take-away doses carries individual and public health risks, these can be minimised by ensuring access to take-home naloxone programmes with naloxone by nasal spray, pre-prepared injection or ampoules of naloxone. Hence, access to take-home naloxone programmes should be rapidly expanded.

Using a risk framework, in combination with assessing the level of patient treatment needs, is one method for stratifying current treatment needs, including the need for supervised OAT dosing. Patients on OAT may have high-level, moderate or low-level treatment needs. This system may assist in deciding the frequency of supervised OAT doses and take-away doses for individuals [19].

It is possible that other providers of opioid treatment, for example, general practitioners and community pharmacies, may not be able to continue to provide opiate treatment throughout the COVID-19 pandemic, due to the need to screen and treat SARS-CoV2, staff illness, or to reduce unnecessary congregation (e.g. in shopping centres or general practices). In this case, state-operated public clinics may be required to enhance their capacity to rapidly assess and treat opioid-dependent patients who can no longer be treated in primary or community care.

Further, additional issues may be faced by patients who must self-isolate for extended periods of time (e.g. 14 days). This, in turn, may require delivery of take-away doses to patient homes, presenting infection control challenges for staff (for example, can we obviate the need for patients to sign that they have received delivery of take-aways by using other means such as a photograph of a patient taking receipt), routine approaches to risk assessment for home visits (as in general, the delivery will not require entry into the person’s home) and ensuring staff as well as patient safety during this period.

OAT service provision in custodial settings may fall under extreme pressure during the COVID-19 pandemic. Many prisons are over-crowded and may struggle to ensure social distancing during this period. Further, it is possible jails may impose ‘lock-downs’ to further limit movement of for security reasons during the pandemic. Daily supervised dosing of methadone and buprenorphine may not remain possible at this time. Transfer to depot buprenorphine should be considered as a way to mitigate this risk and ensure ongoing OAT for people in custody.

### Withdrawal services

Withdrawal services for people who use drugs may also need revision during the COVID-19 pandemic. Withdrawal services may experience additional demand, as people in the community experience interruptions to their use because of reduced supply.

In face of such large scale and unprecedented pandemic prioritised use of intensive and more costly inpatient services should be considered and used only where there is significant medical risk (e.g. risk of severe alcohol withdrawal or alcohol withdrawal seizures or benzodiazepine withdrawal seizures), mental health risk (e.g. significant deterioration of mental health problems) or lack of a suitable home environment to support ambulatory withdrawal. Some hospital inpatient units may be re-purposed by acute hospitals for use as respiratory or other overflow wards. If this is the case, where appropriate, ambulatory withdrawal services should be encouraged to avoid unnecessary admissions and patient congregation in residential or inpatient services. This imposes extra challenges to withdrawal services as additional resources in order to function adequately might be required. As possible measures to be implemented, the use of daily staged medication supply (i.e. daily dosing) during withdrawal should be reconsidered to reduce unnecessary patient travel/attendance at hospital and community pharmacies. In addition, alternative approaches such as increased telephone monitoring and use of carers in the treatment process (e.g. holding medications) may be required. Where opiate agonist treatment can be provided, this should be offered to patients in preference to short-term withdrawal treatment from opioids.

### Relapse prevention medications

Certain treatment models such as the use of disulfiram for alcohol dependence have often been based upon regular attendance at a clinic for monitoring and supervised dosing. Alternative arrangements with increased use of carers, telephone or videoconferencing and monitoring should be explored to reduce the need for travel.

### Hospital drug and alcohol consultation-liaison services

Hospital consultation-liaison (CL) staff may need additional resources to assist emergency departments and acute hospital units in response to COVID-19. Acute services will have to prioritise screening, assessment and treatment for people presenting with respiratory symptoms. Reducing service demand by ensuring people who have acute substance-related problems are efficiently managed (e.g. early management of alcohol withdrawal to prevent complicated alcohol withdrawal/alcohol withdrawal delirium for those at risk) may require an expansion of hospital CL services. Also, avoiding multiple re-presentations of people who use substances and present to hospital emergency departments may be another principal task for CL staff as well as hospital avoidance programmes for people, where these exist.

### Counselling services

Counselling services for people who use drugs may experience changes in demand, either increase or decreases during the COVID-19 pandemic. Counselling services could relatively easily be re-oriented to provide non-face-to-face (i.e. telephone/videoconference) services where possible to minimise patient needs to travel, contact with staff and time in waiting rooms. If there is a reduction in demand for counselling services, these staff may be considered for secondment part or full time to other parts of the service requiring additional staff (e.g. in opiate treatment under surge conditions and providing additional telephone assessment services to assist intake services in meeting demand).

Group counselling should be restricted for only a limited number of participants to facilitate social distancing, or alternately, groups should be cancelled.

### Harm reduction

Minimising the risk of blood-borne virus outbreaks during the COVID-19 period is a priority. Ensuring people who inject drugs continue to have access to clean injecting equipment and other harm reduction services is important. As the demand may increase, services should be prepared to ensure needle syringe and other product availability by providing bulk numbers of needles and syringes (e.g. requests for large supplies 100 syringes and other sterile injecting equipment). Where face-to-face services cannot be provided, vending machines are an efficient method to maintain service delivery around the clock with reduced staffing requirements.

Current harm reduction policies/strategies should be updated and extended so appropriate harm reduction advice can be provided to reduce the spread of COVID-19 and to reduce the consequences of erratic drug markets and supply (e.g. sharing of equipment or drugs such as glass pipes, joints, water pipes (‘bongs’), cigarettes, cash, and straws for ‘snorting’ drugs). Harm reduction services could encourage good hand hygiene by providing patients with education and use of soap and water or hand sanitiser if hand washing is not possible. Hygiene around drug preparation (prepare own drugs) and handling of balloons/baggies (avoiding internal concealment) as well as handling of money should be promoted. As an example of peer harm reduction information, see the INPUD website [20].

Further, opiate users may be at increased risk of overdose given the respiratory impact of SARS-CoV2. People who use opioids could misinterpret SARS-CoV2 flu-like symptoms, including fever (with sweats), aches and pains and fatigue as symptoms of opiate withdrawal. These issues combined could increase overdose risk during this pandemic. People who use drugs by smoking/inhaling may need to consider changing their route of administration (e.g. snorting or rectal use) to reduce the risk of respiratory impact of substance use.

Extended access to take-home naloxone should impose as a key element to be implemented as the risk of opioid overdoses is largely increased due to the current circumstances and described impact in people treatment modalities and access.

### Workplace safety

Principles to inform adjustments to D&A services in the face of an impending pandemic include the following:

1. *Staff health and welfare.* Critical to service continuity for D&A services is to work with frontline staff to ensure that they are supported to continue working, comply with policies to not attend work, home-isolate and be SARS-CoV2 tested if they have respiratory symptoms or meet the criteria for a suspected case. Ensure adequate supplies of personal protective equipment, examine measures to limit staff face to face contact with COVID-19 cases where possible, provide videoconference consultations and avoid travel (e.g. via airports) that expose staff to greater infection risks, identify staff with particularly increased vulnerabilities (pregnancy, chronic diseases, advanced age) and consider redeployment. Staff who live with family at high risk are also presented with the challenge of potentially self-isolation at home or being unable to return to their homes. There is considerable ‘COVID-19 anxiety’ and staff frustration with cancellation of leave or the imposition of additional duties. Consideration of opportunities to improve staff welfare should be a focus where possible particularly if the pandemic becomes protracted. Monitoring of the health of health care workers is important. A study of health care workers in Beijing, China, that followed up staff who worked during the SARS epidemic in 2002/2003 identified an association between health care workers who worked in high-risk locations, such as SARS wards, and subsequent PTSD or alcohol use problems [21].
2. *Clear communication.* Draw on established risk communication principles to keep patients and staff informed as trusted partners [22]. Communicate early, with clarity and transparency, acknowledge current problems, discuss achievements with humility and pay attention to expressed and unvoiced concerns. Ensure communications have a clear call to actions required, as well as what D&A services are doing to manage risk and ensure business continuity.
3. *Early intervention.* The earlier that strategies are enacted to reduce SARS-CoV2 transmission and exposure for both patients and staff, the lesser impact services are likely to experience, enabling service continuity and managing fear and anxiety.
4. *Social distancing, reduced congregation and hygiene/infection control enhancement.* The rationale for this is to reduce the risk of community transmission via confirmed/unconfirmed case contact during this pandemic. This strategy reduces the effective reproductive rate of the pathogen [23]. The principles for this include culture change in terms of standard precautions for infection control, limiting unnecessary gatherings, analysing root causes to prevent congregation and enhance movement of patients through a service. Alternative approaches to service delivery including patient contact by telephone or videoconferencing will reduce the need for travel and reduce the risk of congregation.

## Discussion

Rapid and significant changes will need to occur in treatment services for people who use drugs to ensure optimal service delivery during the COVID-19 pandemic. People who use substances already experience vulnerabilities regarding their medical, mental and social health. The nature of substance use with a high prevalence of tobacco smoking, chronic pulmonary disease, increased overdose risks further increase health problems for this group. Contact with the health care system through access to treatment for people who use drugs is an important way to reduce the risk of worsening substance use-related problems and effective communication of health messages during the pandemic.

Changes in treatment model of care are urgently needed, particularly with OAT to facilitate social distancing. The increased risk of opiate overdose from increased take-away doses of methadone can be reduced by rapid expansion of take-home naloxone. As a non-supervised medication at a population level, buprenorphine-naloxone carries reduced risks of injection [24] and buprenorphine carries reduced overdose risks compared to methadone [25]. Depot buprenorphine, administered by a health care worker, is not associated with a risk of non-medical use [26].

Until either a SARS-CoV2 vaccine or effective medications for COVID-19 have been developed, social distancing is the only possible approach to mitigate the community impact and reduce viral spread and deaths from COVID-19 [27]. Treatment services for people who use drugs must re-configure to assist this.

### Ethics

Key ethical considerations during the COVID-19 affecting people who use drugs and need to access treatment include the issue that people who use drugs may prioritise using substances (e.g. to avoid substance withdrawal) above their personal safety (e.g. to follow country recommendations for home isolation). During Hurricane Katrina, there are reports of some people who use drugs prioritising access to and use of drugs above their personal safety and ignored health and safety messages [28].

During the COVID-19 pandemic, it is possible that some people with substance use problems may prioritise drug use and the need to ensure they receive treatment ahead of other concerns, such as advising treating staff of symptoms of an illness/acute SARS-CoV2 symptoms, avoiding congregation and isolation or treatment when this should occur. Ensuring ongoing access to drug treatment for all those who need treatment and may limit this risk but other mitigation strategies may be required.

Escalated use of violence has been described during hurricanes Katrina, Ike and Gustav in the USA related to rapidly changing situations for people who use drugs including drug availability and the risks of buying and selling illicit drugs during and after a weather-induced disaster [29]. Changes to drug supply and risks for people who use drugs during COVID-19 may not be as rapid nor volatile but may be more long-lasting. It is important services remain alert to the risks for people who use drugs during this period.

Stigma and discrimination towards people who use drugs is sadly a commonly described occurrence. During times of crisis on the health care system, marginalised groups including people who use drugs are particularly at risk of not being able to access health care or not receive the same quality of health care as others in the community due to poorer health literacy among this group and biassed perceptions by healthcare providers. People who are homeless are particularly vulnerable during this period. Clinicians play an important role in advocating for people who use drugs who access the health care system.

There should be a primary principal of equity of access to treatment, both treatment for substance use problems generally and respiratory screening and treatment where required, for people who use drugs regarding SARS-CoV2 infection. It is possible not all patients attending treatment services for substance use problems will comprehend the health messages, as health literacy in this group is low. Treatment service providers have an important role in working with patients who attend services in encouraging co-operation with public health messages and recommendations.

### Health messages for patients

Providing clear health information for people who use drugs needs to occur during the COVID-19 pandemic. Access to and availability of treatment services may change, so this should be communicated clearly and with as much notice as possible. Health literacy may be poor in this group, so easy to understand messages, using diagrams and plain infographics could assist in delivering clear messages. Staff will need to spend additional time with patients to reduce misunderstanding and improve knowledge during this period.

It is important clinicians are aware of this issue and discuss this with patients, so opiate withdrawal and COVID-19 are not confused leading to misdiagnosis, late presentation and community spread of the virus.

## Conclusion

People who use drugs and or access treatment services for substance use may be particularly at risk during the current COVID-19 pandemic. As acute health care systems are expected to experience an unprecedented demand on health care services during this period, ensuring people with substance use problems continue to receive services may create numerous challenges during this time.

Treatment services for people who use drugs may need to rapidly review how and to whom services are provided, with the capacity for expansion of some services to meet surge demand, re-purposing of services and secondment of staff. Providing stable opiate agonist treatment, access to injecting equipment and other key treatment services for people who use drugs should remain a priority during the period COVID-19 affects local communities.

## Data Availability

Not applicable
